# Efficacy of attachment-based family therapy compared to treatment as usual for suicidal ideation in adolescents with MDD

**DOI:** 10.1177/1359104520980776

**Published:** 2020-12-21

**Authors:** Luxsiya Waraan, Erling W Rognli, Nikolai Olavi Czajkowski, Lars Mehlum, Marianne Aalberg

**Affiliations:** 1 Division of Mental Health Services, Akershus University Hospital, Lørenskog, Norway; 2 Department of Psychology, University of Oslo, Oslo, Norway; 3 Department of Child and Adolescent Mental Health Services, Akershus University Hospital, Lørenskog, Norway; 4 PROMENTA Research Center, Department of Psychology, University of Oslo, Oslo, Norway; 5 Department of Mental Disorders, Norwegian Institute of Public Health, Oslo, Norway; 6 National Centre for Suicide Research and Prevention, Institute of Clinical Medicine, University of Oslo, Oslo, Norway

**Keywords:** Brief report, suicidal ideation, family Therapy, ABFT, adolescent

## Abstract

Attachment-Based Family Therapy (ABFT) is the only empirically supported family therapy model designed to treat adolescent depression, including those at risk for suicide, and their families. ABFT aims to repair interpersonal ruptures and rebuild an emotionally protective parent-child relationship. To study the effectiveness of ABFT compared with Treatment as Usual (TAU) in reducing suicidal ideation in clinically depressed adolescents. Sixty adolescents (86.7% girls), aged 13 to 18 years (*M* = 14.9), with major depressive disorder referred to two CAMHS were randomized to receive 16 weeks of ABFT or TAU. ABFT consisted of weekly therapy sessions according to the treatment manual. Suicidal ideation was measured with the Suicidal Ideation Questionnaire-Junior at 4, 6, 8, 10, 12, 14, and 16 weeks. Linear mixed models were fitted to test our hypothesis, time was the only factor to have a significant effect on suicidal ideation *t*(31.05) = −3.32, *p* < .01. Participants in both treatment groups reported significantly reduced suicidal ideation, but the majority were still in the clinical range after 16 weeks of treatment. ABFT was not associated with more favorable outcomes than TAU. Findings must be interpreted with caution given the study limitations.

Suicide is one of the leading causes of death in adolescents worldwide (Glenn et al., 2020; WHO, 2020). One of the strongest risk factors for death by suicide or suicide attempt is depressive disorder accompanied by suicidal ideation (Beghi et al., 2013). Despite the high prevalence of suicidal thoughts and behaviors among youth (Casey et al., 2008; Nock et al., 2013), there is limited knowledge about interventions that are effective in reducing suicidal ideation in adolescents with Major Depressive Disorder (MDD).

Cognitive behavioral therapy and Interpersonal psychotherapy have emerged as well-established treatment approaches for youth with MDD (Eckshtain et al., 2020; Weersing et al., 2017). Evidence-based psychotherapies for depression are also included in treatment guidelines for suicidality, however, there is limited evidence as to whether these treatments reduce suicidal ideation (Cuijpers et al., 2013). Continued efforts to improve treatment for these youth are warranted and one promising direction is family therapy. Family relationships can both maintain symptoms of depression, including suicidal ideation, and serve as a protective factor. Increased family focus may be one pathway to improving the effect of psychotherapy for depressed youth with suicidal ideation. Among treatment approaches with a family focus is Attachment Based Family therapy (ABFT), which has been shown to be efficacious in reducing suicidal ideation (Diamond et al., 2010, 2018). The main assumption for this approach is that a negative family environment inhibits children from developing the internal and interpersonal coping skills needed to buffer against family, social, and community stressors. ABFT sessions focus on rebuilding trust and communication with parents and helping parents become better caregivers.

In a recent study, designed to examine the effectiveness of ABFT compared to Treatment as usual (TAU) in reducing depressive symptoms, we found no differences between the two treatment options (Waraan et al., 2020). Neither ABFT nor TAU was efficient in reducing depressive symptoms. In the present report we compare the efficacy of the two treatments in reducing suicidal ideation in adolescents with MDD. Because ABFT has been shown to be especially efficient in reducing suicidal ideation, we hypothesized that patients receiving ABFT would show greater reduction in suicidal ideation than adolescents receiving TAU.

## Method

**Figure 1.**
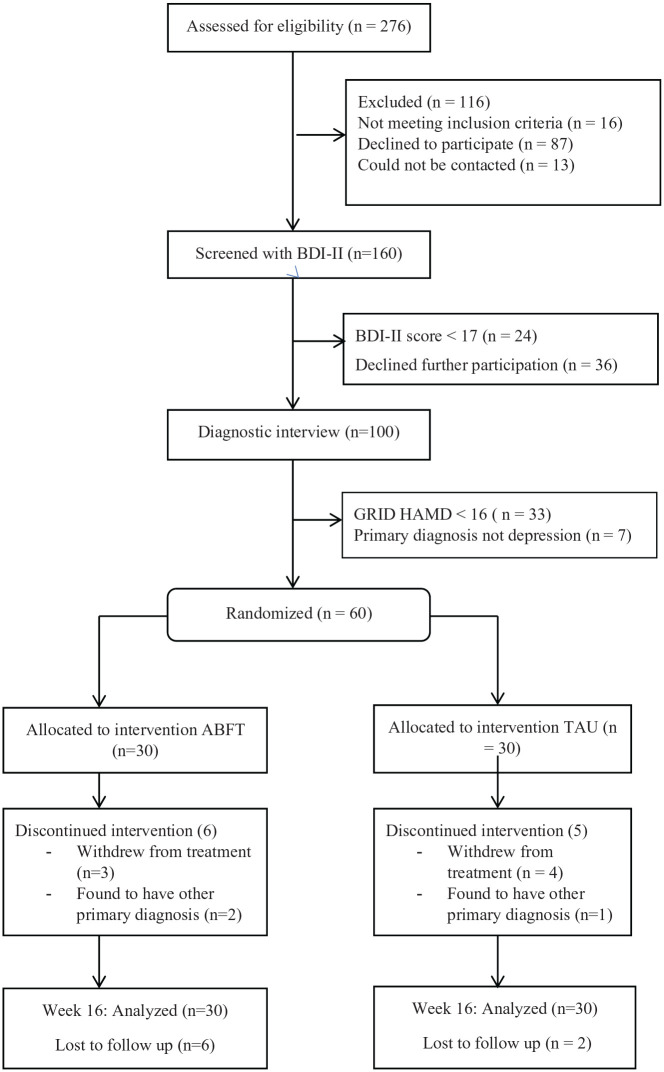
Consolidated Standards of Reporting Trials (CONSORT) flowchart of participants comparing Attachment Based Family Therapy (ABFT) with Treatment as Usual (TAU). *Note*. BDI-II = Beck Depression Inventory – II, GRID-HAMD = GRID – Hamilton Depression Rating Scale.

This is a secondary analysis of data from a 16-week randomized controlled trial of adolescents with depression (www.clinicaltrials.gov NCT01830088) conducted at two Child and Adolescent Mental Health Service (CAMHS) clinics in the county of Akershus, Norway. The study was approved by the regional committee for medical research ethics, South-Eastern Norway. The study methods have been described in detail in a previous publication (Waraan et al., 2020). Briefly, participating adolescents and their families were recruited among adolescents referred to two CAMHS clinics in South-Eastern Norway. Inclusion criteria were (1) a diagnosis of current major depressive episode, based on a semi structured diagnostic interview, and (2) a score above 15 on the Grid Hamilton Depression Rating scale (GRID-HAMD, Williams et al., 2008). The exclusion criterion was a diagnosis of any psychotic disorder, eating disorder, bipolar disorder, intellectual disability or pervasive developmental disorder. Eligible adolescents and parents provided written, informed consent and were randomized to either ABFT or TAU. Randomization was stratified by clinic, age (13–15 years and 16–18 years), gender, and severity of depression (GRID-HAMD score of ⩽24 and ⩾25). The randomized study sample consisted of 60 participants, 30 in each treatment condition. See Figure 1 with Consolidated Standards of Reporting Trials (CONSORT) flowchart for progression through the study. Treatment consisted of 16 weeks of either ABFT or TAU.

## Assessment

For the duration of the treatment patients completed self-report measures electronically every other week using a secure online platform (CheckWare Assessment Systems). Some self-report measures were administered as paper and pencil questionnaires by the treating clinicians as some participants experienced technical difficulties with the online forms. The Schedule for Affective Disorders and Schizophrenia for School-Age Children Present and Lifetime Version (K-SADS-PL) (Kaufman et al., 1997) interview was conducted at baseline for the adolescent and parents separately. Interrater reliability was determined by blind scoring of 28 randomly selected videotaped interviews. Interrater reliability for MDD based on the K-SADS interview was κ = .56.

Suicidal ideation was measured with the Suicidal Ideation Questionnaire-Junior (SIQ-Jr) (Reynolds, 1988). SIQ-Jr is a 15-item self-report questionnaire measuring frequency and severity of suicidal thoughts on a 7-point scale with a total score ranging from 0 to 90 with higher scores indicating more suicidal thoughts. A score of 31 is recommended as clinical cut off (89th percentile of the normative sample), a score above this level is indicative of elevated suicidal risk. Internal consistency in the current sample was α = .95. SIQ-JR was completed at baseline and then every other week from week 4 to end of treatment at week 16.

## Data analytic strategy

SIQ-JR had an average of 62 % missingness during the 16 weeks of treatment (see appendix 1). In some cases, adolescents actively declined to provide data. In other cases, participants did not manage to complete the self-reported measures electronically. Due to lack of capacity and resources, participants were not targeted for renewed attempts to collect their data. Statistical analyses were conducted using SPSS Version 23 (IBM Corp, 2016) for Windows. SIQ-Jr was analyzed in a mixed modeling framework, handling missing observations using maximum likelihood estimation (Enders, 2010). As a first step we evaluated competing models of the effect of time on suicidal ideation, independent of treatment (models 1a–1c). Subsequently, treatment effect was tested in two steps. First we included a main effect of treatment group, which allows for a difference on the intercept at baseline across the two groups (2a), before we finally added a time*treatment group term, which allows the expected levels of suicidal ideation to differ across time in the two treatment groups (2b) see Table 2. Of the five models tested, the model with the lowest values on the information criteria (AIC/BIC) was selected as the overall best fitting one. Data were analyzed by intent-to-treat principles.

## Results

**Figure 2.**
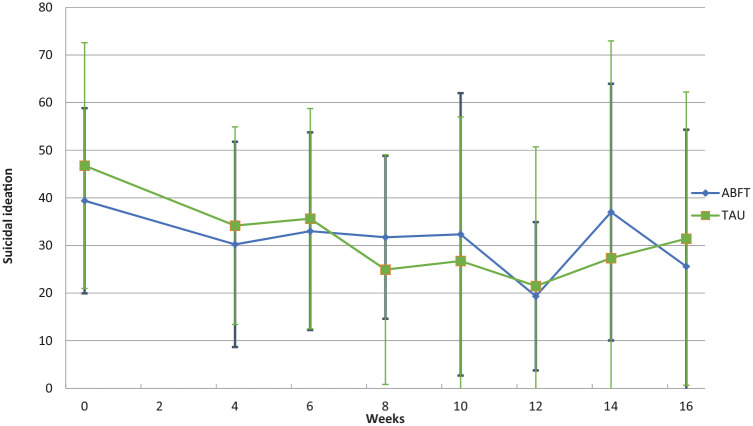
Self reported Suicidal Ideation by treatment condition at baseline and after 4, 6, 8, 10, 12, 14, and 16 weeks of treatment. *Note*. Error bars indicate standard deviation.

Table 1 summarizes the baseline sociodemographic data and clinical characteristics of the participants by treatment condition. Both treatment groups had an average baseline severity of suicidal ideation well above the clinical cut-off. Suicidal ideation was analyzed through a set of mixed models. The linear mixed models were fitted to test whether treatment condition was significantly related to change in scores over time. In models 1a to 1c, the effect of time on suicidal ideation was tested. Model 1c with both a fixed and random effect of time was the best fit for the data. In the next models, treatment condition was added as a fixed effect. Model 2a with both a fixed and random effect of time, and treatment as a fixed effect was the best fit for the data with the least IC (AIC = 564.18, BIC = 576.95) (see Table 2). Time was the only factor to have a significant effect on suicidal ideation, *t*(31.05) = −3.32, *p* < .01. The fixed effect of treatment allocation was not significantly associated with change in suicidal ideation scores over time. All random effects were significant across all the models. Both treatment groups showed similar reductions in suicidal ideation over the first few weeks (see Figure 2) and reported the lowest level of suicidal ideation at week 12, while at the end of treatment adolescents in both groups experienced an increase in suicidal ideation.

**Table 1.** Baseline demographic and diagnostic data in adolescents (*N* = 60) allocated to receive 16 weeks of Attachment Based Family Therapy (ABFT) or Treatment as Usual (TAU).

Variable		Treatment condition	
		ABFT (*n* = 30)	TAU (*n* = 30)
Age, years (*SE*)		15.03 (1.35)	14.77 (1.36)
Gender, % (*n*)	Female	90 (27)	83.3 (25)
Dropout, % (*n*)	Excluded	7 (2)	3.3 (1)
	Drop out	10 (3)	13.3 (4)
Ethnicity, % (*n*)	Norwegian	100 (30)	96.7 (29)
	Scandinavian other than Norwegian	0 (0)	3.3 (1)
Living with, % (*n*)	Both parents	29.6 (8)	36.7 (11)
	Two home family	18.5 (5)	13.3 (4)
	Father (and partner)	18.5 (5)	13.3 (4)
	Mother (and partner)	33.3 (9)	33.3 (10)
	Other/missing	10 (3)	3.3 (1)
Psychiatric comorbidity, % (*n*)	Dysthmia	3.3 (1)	0 (0)
	Any anxiety disorder^a^	43.3 (13)	46.7 (14)
	Obsessive-Compulsive Disorder	6.7 (2)	6.7 (2)
	Externalizing disorder^b^	0 (0)	13.4 (4)
	PTSD	3.3 (1)	3.3 (1)
	Eneuresis	3.3 (1)	6.7 (2)
	No comorbidity	53.3 (16)	46.7 (14)
Suicidal ideation (SIQ-Jr), mean (*SD*)		39.40 (19.45)	46.77 (25.81)
Non suicidal Self-harm^c^		60 (18)	70 (21)
Self harm		50 (15)	23.3 (14)
Suicide attempt^d^		30 (9)	33 (10)

% (*N*) unless stated otherwise, SIQ-JR = Suicidal Ideation Questionnaire – Junior.

a Includes social phobia, specific phobia, agora phobia, generalized anxiety disorder, Anxiety INA, obsessive compulsive disorder.

b Includes oppositional defiant disorder, attention deficit/hyperactivity disorder.

c Non Suicidal Self harm – Includes with and without intention to die.

d Self-harm behavior with an explicit or inferred intention to die.

**Table 2.** Linear mixed model of self-reported suicidal ideation measured at baseline and after 4, 6, 8, 10, 12, 14, and 16 weeks.

Fixed effect	Null model			Model 1a			Model 1b			Model 1c		
	*b*	CI	*p*	b	CI	*p*	*b*	CI	*p*	*b*	CI	*P*
Intercept	2.48	[2.09, 2.86]	.00***	2.74	[2.35, 3.13]	.00***	2.86	[2.46, 3.25]	.00***	2.76	[2.37, 3.15]	.00***
Time				−.06	[–.09, –.03]	.00***	−.15	[–.23, –.08]	.00***	−.07	[–.11, –0.03]	.00**
Time^2^							.01	[.001, .01]	.01**			
Random effects												
σ^2^_Intercept_	1.83*			1.74**			1.74***			1.89**		
σ^2^_Time_										.01*		
Residual	.75*			.66**			.64***			.46**		
Information Criteria												
AIC	587.86			575.82			579.36			564.64		
BIC	594.27			582.22			585.74			577.44		
Log-Likelihood	583.86			571.82			575.35			556.64		

**Table:** 

Fixed effect	Model 2a			Model 2b		
	*b*	CI	*p*	*b*	CI	*p*
Intercept	2.91	[2.37,3.45]	.00***	3.02	[2.47,3.58]	.00
Time	−.07	[–.11, –.03]	.00**	−.10	[–.16,–.05]	.002**
ABFT	−.30	[–1.04, .44]	.42	−.53	[–1.31, .25]	.18
ABFT × Time				.07	[–.01, .16]	.07
Random effects						
σ^2^_Intercept_	1.87***			1.86***		
σ^2^_Time_	.01*			.01*		
Residual	.46***			.46***		
AIC	564.18			565.38		
BIC	576.95			578.12		
Log-Likelihood	556.18			557.38		

*Note*: ABFT = Attachment Based Family Therapy.

* *p* ⩽ .05. **p ⩽ .01. ***p ⩽ .001.

## Discussion

In the present study, level of suicidal ideation decreased with treatment, but there was no significant difference between participants in the ABFT and TAU conditions. The lack of differences between ABFT and TAU is consistent with findings by Diamond et al. (2018). Previous studies have, however, reported greater reductions in suicidal ideation after 16 weeks of ABFT than what we find in our study. Levels of suicidal ideation fluctuated from week to week. At end of treatment, the majority of adolescents in this study still reported a substantial level of suicidal ideation. Our finding is in line with a recent review by Glenn et al. (2019), where ABFT was downgraded from probably efficacious to an experimental intervention for reducing suicidal ideation in adolescents. Considering a recent report by Diamond et al. (2018), in which more than half of the sample did not achieve remission in suicidal ideation, taken together with previous findings one may infer that the attachment-based components rooted in ABFT do not result in a superior psychotherapy, contrary to prevailing beliefs.

Family involvement has become a cornerstone in treatment of children and adolescents. In ABFT one assumption is that insecure attachment to parents or caregivers can exacerbate suicidal ideation and that there is a relational rupture that needs repair (Hunt et al., 2017). Adolescence is a period of social reorientation, in which social influences expand beyond the family to emphasize peers. Exposure to peer stress and peer victimization has been shown to be associated with suicidal ideation (Prinstein et al., 2000; van Geel et al., 2014). Interpersonal relationships with peers and interpersonal skills may therefore be equally important as family relationships as a focus in psychotherapy. In our study, there was a significant reduction in suicidal ideation over time, but adolescents in both groups still reported suicidal ideation at a clinical level (TAU) or just below (ABFT) at end of treatment, indicating that adolescents in both groups still were in distress.

ABFT was originally developed as a therapy method for adolescents with depression. Whether psychotherapies, like ABFT, designed for the treatment of depression reduce suicidal ideation more than TAU is at best unclear. The continued debate on the mutual dependence between depression and suicide is important to consider, as it will affect the way clinicians work with suicidal ideation. Traditionally, suicidal ideation has been understood as a symptom and indicator of underlying mental disorder, and consequently researchers suggested that treatments that reduce depressive symptoms, also reduce suicidal ideation (Weitz et al., 2014). However, others have questioned this belief, and suggested that suicidal ideation and depression might be related, but are independent constructs (Oquendo & Currier, 2009) and that hopelessness may be the mediator between them (Cuijpers et al., 2013). Psychotherapy for depression may have a small positive effect on suicidal ideation, but may be insufficient. Suicidal ideation should be directly addressed and targeted. Suicide specific treatment models may be more effective in reducing suicide risk and suicidal behavior. This could be one of the reasons why there is only one empirically supported intervention, Dialectical Behavior Therapy (DBT-A) (Mehlum et al., 2014; Miller et al., 1997) evaluated as a well-established intervention for reducing suicidal ideation in adolescents (Glenn et al., 2019). DBT (Linehan, 1993) was developed for highly suicidal emotionally dysregulated adults with borderline personality disorder (BPD), and adapted for adolescents, with good results (McCauley et al., 2018; Mehlum et al., 2014, 2016, 2019). Considering suicidal ideation only as a symptom of MDD or BPD, as described in the DSM-V (American Psychiatric Association, 2013) may lead clinicians to overlook suicidal ideation in the context of other psychiatric disorders, and underestimate the patient’s level of suicidal ideation. On the other hand, the vast majority of adolescents with suicidal ideation meet criteria for at least one psychiatric disorder (Nock et al., 2013). There is clearly an association between mental disorders and suicidal ideation (Cash & Bridge, 2009; Nock et al., 2009). Hence, looking at suicidal ideation as a dimension completely separate from psychiatric disorders may not be fruitful. Adolescents struggling with suicidal ideation present with high levels of heterogeneity, complexity and co-occurring problems (Pompili, 2019). There is a need to directly address suicidal ideation in the context of psychiatric disorders and establish methods to reliably identify young people without mental disorders with suicidal ideation who are at risk. One third of adolescents who have suicidal ideation go on to make a suicide plan, and approximately one third of them will make a suicide attempt (Nock et al., 2013). Most of the adolescents who make the transition from suicidal ideation to suicidal behavior will do so within 1 year after the onset of suicidal ideation (Nock et al., 2013), which highlights the continued need for efficient identification and treatment for these vulnerable youths.

The results from the present study should be interpreted in the context of its limitations and strengths. Our study was underpowered and the missingness on the outcome of interest, suicidal ideation, was high. Additionally, there were no fidelity assessments and TAU was not monitored. However, the fact that the study was conducted in a community mental health setting with patients recruited from a defined catchment area, strengthens the external validity of the findings. A failure to report less than ideal results can lead to inefficient treatments being implemented. Findings that do not support the assumed efficacy of an intervention, need to be transparently reported to give a clear picture of the research field. Our results could be an indication that suicidal ideation in adolescents with depressive disorder needs to be addressed more directly and specifically. Finally, we emphasize the need for future studies to replicate promising interventions by independent research groups.

## Supplemental Material

sj-pdf-1-ccp-10.1177_1359104520980776 – Supplemental material for Efficacy of attachment-based family therapy compared to treatment as usual for suicidal ideation in adolescents with MDD Click here for additional data file. Supplemental material, sj-pdf-1-ccp-10.1177_1359104520980776 for Efficacy of attachment-based family therapy compared to treatment as usual for suicidal ideation in adolescents with MDD by Luxsiya Waraan, Erling W Rognli, Nikolai Olavi Czajkowski, Lars Mehlum and Marianne Aalberg in Clinical Child Psychology and Psychiatry
