# Supplementary material for: Efficacy of attachment-based family therapy compared to treatment as usual for suicidal ideation in adolescents with MDD
Source: Clin Child Psychol Psychiatry. 2020 Dec 21;26(2):464–74. doi: 10.1177/1359104520980776 (PMC8041448; doi:10.1177/1359104520980776)
Supplement: sj-pdf-1-ccp-10.1177_1359104520980776 – Supplemental material for Efficacy of attachment-based family therapy compared to treatment as usual for suicidal ideation in adolescents with MDD [file sj-pdf-1-ccp-10.1177_1359104520980776.pdf]

Table 3: Suicidal Ideation (measured by SIQ) distribution from baseline to week 16.

		Baseline	Week 4	Week 6	Week 8	Week 10	Week 12	Week 14	Week 16
ABFT	N (%)	30 (100)	15 (50)	18 (60)	8 (26.67)	6 (20)	6 (20)	4 (13.33)	5 (16.67)
	M (SD)	2.63 (1.30)	2.02 (1.44)	2.20 (1.38)	2.11 (1.14)	2.16 (1.98)	1.29 (1.04)	2.47 (1.80)	1.71 (1.91)
TAU	N (%)	29 (96.67)	12 (40)	16 (53.33)	8 (26.67)	8 (26.67)	8 (26.67)	3 (10)	7 (23.33)
	M (SD)	3.12 (1.72)	2.28 (1.38)	2.38 (1.54)	1.66 (1.61)	1.78 (2.02)	1.43 (1.95)	1.82 (3.04)	2.10 (2.05)
